# Warm and humidified insufflation gas during gynecologic laparoscopic surgery reduces postoperative pain in predisposed patients—a randomized, controlled multi-arm trial

**DOI:** 10.1007/s00464-021-08742-1

**Published:** 2021-10-01

**Authors:** Markus Breuer, Julia Wittenborn, Rolf Rossaint, Julia Van Waesberghe, Ana Kowark, Deborah Mathei, András Keszei, Svetlana Tchaikovski, Magdalena Zeppernick, Felix Zeppernick, Elmar Stickeler, Norbert Zoremba, Ivo Meinhold-Heerlein, Christian Bruells

**Affiliations:** 1 Department of Anesthesiology, University Hospital of the RWTH Aachen, Pauwelsstrasse 30, 52074 Aachen, Germany; 2 Department of Gynecology and Obstetrics, University Hospital of the RWTH Aachen, Pauwelsstrasse 30, 52074 Aachen, Germany; 3 Department of Medical Statistics, University Hospital of the RWTH Aachen, Pauwelsstrasse 30, 52074 Aachen, Germany; 4 Department of Gynecology and Obstetrics, University Hospital of Gießen and Marburg, Justus-Liebig University Gießen, Klinikstr. 33, 35392 Giessen, Germany; 5 Department of Anesthesiology and Intensive Care, St Elisabeth Hospital, Stadtring Kattenstroth 130, 33332 Gütersloh, Germany

**Keywords:** Laparoscopy, Gynecology, Postoperative pain, Warm humidified insufflation gas

## Abstract

**Background:**

Postoperative pain remains a common problem in gynecologic laparoscopy, especially in head zone-related regions, triggered by intra-abdominal pressure during capnoperitoneum. Humidified and prewarmed insufflation gas may ameliorate pain and be beneficial.

**Methods:**

This prospective randomized controlled parallel group multi-arm single-center study investigated the effects of temperature and humidity of insufflation gas on postoperative pain during gynecologic laparoscopy with a duration ≥ 60 min. Female participants (18—70 years) were blinded and randomly assigned—computer generated—to either insufflation with dry cold CO_2_ with forced air warming blanket (“AIR”), humidified warm gas without forced air warming blanket (“HUMI”), or humidified warm gas with forced air warming blanket (“HUMI +”). We hypothesized that using humidified warm gas resulted in lower pain scores and less analgesic consumption. The primary endpoint postoperative pain was assessed for different pain localizations every 12 h during 7 days after surgery. Secondary endpoints were demand for painkillers and epidural anesthetics, length of stay in recovery room, and hospital stay. (Registration: ClinicalTrials.gov NCT02781194—completed).

**Results:**

150 participants were randomized. Compared to group “AIR” (*n* = 48), there was significantly less pain in group “HUMI +” (*n* = 48) in the recovery room (− 1.068; 95% CI − 2.08 to − 0.061), as well as significantly less ibuprofen use at day two (− 0.5871 g ± 0.258; *p*-value = 0.0471). Other variables did not change significantly. Stratification for presence of endometriosis or non-previous abdominal surgery in patient history revealed significantly less pain in both groups “HUMI” (*n* = 50) and “HUMI +” versus group “AIR.” Related side effects were not noted.

**Conclusion:**

In the overall population, the use of warm, humidified insufflation gas did not yield clinically relevant effects; however, in predisposed patients with endometriosis and who could otherwise expect high pain levels, warm and humidified gas may be beneficial.

**Supplementary Information:**

The online version contains supplementary material available at 10.1007/s00464-021-08742-1.

Since its invention, laparoscopic surgery became the gold standard in various surgical disciplines with a wide range of indications [1]: the majority of gynecological surgeries are currently performed using minimally invasive technique due to its benefits compared with open access. Patients undergoing laparoscopic surgery benefit from a faster recovery, a reduced hospital stay, and a quicker return to normal activities [1, 2], resulting in increased patient satisfaction [3]. Until 2018, the frequency of laparoscopic appendectomy, cholecystectomy, and hysterectomy was reported to have increased worldwide [4].

Despite these benefits and the rising numbers of laparoscopic procedures, postoperative pain remains a common problem [5]. Gerbershagen et al. showed that unexpectedly high levels of postoperative pain occur even in some minor-to medium level surgical procedures using the laparoscopic approach [6]. In addition to wound-related pain, up to 80% of patients undergoing laparoscopic procedures also complain about shoulder tip pain [7] that is often perceived to be more hampering and disabling than the wound pain. The severity of the postoperative pain is dependent not only on the type and conditions of the surgical procedure but is also influenced by the preoperative patient characteristics. For instance, the severity of pre-existing dysmenorrhea, a common symptom of endometriosis [8], predicts significantly higher levels of postoperative pain [5], which results in patients’ discomfort, a longer stay in the hospital, and higher consumption of analgesics, thereby increasing the frequency of their side effects [9, 10].

There are several approaches proposed to reduce postoperative pain, especially shoulder tip pain, in gynecological laparoscopy. One of the proposed methods is the use of warmed and humidified carbon dioxide (WHCD) for establishing the capnoperitoneum. Animal studies have shown that the use of WHCD during laparoscopy results in less desiccation and cell alteration and therefore less peritoneal damage and inflammation as compared to cold and dry gas [11–13]. Dry gas causes peritoneal tissue drying, cell death, and the loss of peritoneal surface continuity [14]. There is also evidence for a rapid and significant induction of HIF-1α by cold and dry CO_2_ compared with WHCD [15].

Consequently, Binda concluded in her review that both pain and tissue damage can be prevented using humidified gas [16]. However, recent meta-analyses investigating the effects of WHCD on postoperative pain report contradictory results due to the small sample size of the available studies, varying duration of operation time, and the comparison of different surgical procedures, e.g., visceral and gynecological [17–19]. One key factor of the STP incidence after laparoscopy seems to be the duration of the surgical procedure per se [5, 20].

We therefore decided to investigate the impact of WHCD on postoperative pain course following gynecological laparoscopic procedures with a duration of more than 60 min in a prospective, randomized, controlled monocentric multi-arm trial (A prospective, randomized, controlled, double-blinded study investigating intraoperative temperature and postoperative pain course following gynecological laparoscopy—TePaLa (Temperature and Pain in Laparoscopy)). This article describes parts of this TePaLa trial (the effects on body temperature have not been published yet). The TePaLa trial is based on a retrospective pilot study showing the preventive effect of body temperature and humidified CO_2_ on intraoperative hypothermia compared to room temperature and dry gas in laparoscopy that lasted at least 60 min [21]. As perception of postoperative pain was likely to be influenced by pre-existing endometriosis, the data were stratified for this disorder.

We hypothesized that using WHCD compared to cold and dry carbon dioxide resulted in lower pain scores, especially shoulder pain, and less analgesic consumption. Since patients suffering from endometriosis are more prone to having severe postoperative pain, we suggested that they could profit more from this kind of insufflation technique as compared to women who underwent surgery because of other gynecological diseases. In a combined three-arm study design investigating pain and temperature management, patients were either warmed with a forced air warming blanket, with the use of WHCD, or with a combination of both.

The aim of this section of the study was to assess the impact of forced air warming or WHCD on postoperative pain course following gynecological laparoscopic procedures with a duration of more than 60 min.

## Methods

### Trial design

The study was designed as a monocentric, prospective, randomized, double-blinded controlled trial with three parallel intervention arms. Before trial commencement, the study design was changed to be a single-blinded trial because the surgeons and study staff could not be effectively blinded with respect to the devices used during the laparoscopic procedure. All patients and ward staff were not aware of the method used during laparoscopy.

The methods and trial are described in detail in supplement 2.

### Participants

The study included 150 participants with an indication to a laparoscopic gynecological surgery. It was conducted at the Department of Anesthesiology and the Department of Gynecology and Obstetrics, University Hospital Aachen, Germany between July 2016 and September 2018.

The participants were randomized in 3 groups of 50 subjects each. In group “AIR” (control group “AIR”), a forced air warming blanket and cold and dry insufflation gas was used during surgery. In group “HUMI”, insufflation was performed with warm and humidified insufflation gas and no warming blanket was used (“HUMI”). Group “HUMI +” was treated with a combination of a forced air warming blanket and warm, humidified gas (“HUMI +”).

### Inclusion criteria

Eligible patients were female, aged between 18 and 69 years with a body mass index under 35, admitted to the hospital for laparoscopic surgery with a planned duration of more than 60 min.

### Exclusion criteria

Exclusion criteria were patients who were pregnant or not using sufficient contraception, who were breastfeeding, who were engaged in alcohol or drug abuse, who were either expected not to comply with instructions or with limited ability to comply with instructions for this study, who were unwilling or unable to give informed consent, who participated in another interventional study within the last 3 months, who are committed to an institution and/ or penitentiary by judicial or official order, and who are employees of the investigator cooperation companies.

### Interventions

#### Intraoperative procedures

If epidural anesthesia was indicated and desired by the patient, an epidural catheter was placed according to standard operating procedures. All patients received general anesthesia as total intravenous anesthesia or low flow (< 1 l/ min)-balanced anesthesia. After the induction of anesthesia, patients of group “AIR” and group “HUMI +” received forced air warming and patients of group “HUMI” were only covered with cotton sheets. According to randomization, capnoperitoneum was established and maintained either with cold and dry CO_2_ (21.0 °C room temperature/ 0% humidity) in group “AIR” or with warm and humidified CO_2_ (depending on flow rate > 38.6 °C/ > 98%) [22] in group “HUMI” and group “HUMI +”. The actively heated tube maintained the temperature and humidity of the gas until it was delivered to the patient interface (37.0 °C ± 0.8/ 100.0% ± 0.05) [23].

#### Post-surgical data acquisition

After the patient’s arrival in PACU, the pain score was determined with the visual analogue scale (VAS) for pain from the abdominal area, pain in the shoulder, pain upon movement, and pain upon coughing. Pain scores were also recorded before transfer to the ward, on the day of surgery at 8 p.m., and on postoperative days 1 to 7 at 8 a.m. and 8 p.m. until the day of the patient’s dismissal from the hospital. All patients were instructed to use VAS on the day before surgery, and the pain questionnaire was filled out by the patient alone to avoid observer bias. Postoperative pain management was standardized and followed a three-step analgesic ladder, based on the WHO guidelines for the pharmacological and radiotherapeutic management of cancer pain in adults and adolescents [24].

### Outcomes

The primary endpoint was postoperative pain recorded by the visual analogue scale upon arrival in the recovery room, before transfer to the ward, at 8 p.m. on the day of surgery, at 8 a.m. and 8 p.m. on postoperative days 1 to 7 specifically for abdominal pain, pain in the shoulders, pain upon movement, and pain upon coughing. Secondary endpoints were analgesic consumption, the duration of epidural anesthesia, postoperative nausea and vomiting, differences in activities of daily living (ADL), the length of stay in post-anesthesia care unit (PACU), and the total length of the hospital stay.

### Sample size

This study was designed to address heating capabilities and pain reduction. Three groups were constructed, and sample size and statistical power were calculated to detect a difference in core body temperature. In a Cohen's delta effect size power analysis, a sample size of 50 in each of the treatment groups would give a power of 0.8 to detect a difference of at least 0.2 °C between groups in a balanced design. The effects on body temperature have not been published yet.

### Randomization

After patients were enrolled by the study team and written informed consent was obtained, study participants were randomized with equal allocation ratios to the three interventions using permuted block randomization (block size 6) stratified by endometriosis (Yes/No). Computer-generated sequences were used. To maintain allocation concealment, the randomization sequence and the block size were concealed from the investigators and the study team until database lock and the assignment to study participants was carried out with a web-based application maintained by the Institute of Medical Informatics, RWTH Aachen University.

### Statistical methods

Outcome variables were described within each treatment group using standard descriptive statistics (frequency, minimum, maximum, quartiles, mean, and standard deviation). Descriptive statistics for pain scores were also calculated separately for each measurement timepoint. Analyses of pain scores were performed on the maximum pain score calculated as the maximum of the abdominal-, movement-, upon coughing-, and shoulder pain scores for each subject at each measurement occasion. A linear mixed effects model was used to model the pain score [25, 26]. Estimated treatment effects at each measurement occasion were calculated from the model along with nominal 95% confidence intervals. Explorative tests for the treatment effect on activities of daily living (ADL) scores and the frequency of nausea severity levels on day 1 and on discharge day were conducted with Kruskal–Wallis rank sum test and Pearson’s Chi-squared tests, respectively. The length of the stay in PACU was analyzed using a general linear model with gamma-distributed errors. Analyses were conducted using R [27]. Mixed models were fitted with lme4 [28].

## Results

### Study population

A total of 208 patients with an indication to a laparoscopic gynecologic surgery were assessed for eligibility between July 2016 and September 2018. The trial ended after the planned 150 interventions were completed. 58 subjects were not included, as they did not meet inclusion criteria, declined to participate, participated in another study, or for other reasons. The 150 patients who were included in the study were randomized either to the control group or to one of the two intervention groups, stratified by endometriosis. Seventy-four women declared to suffer from endometriosis and 76 did not have a diagnosis of endometriosis. Four of these patients were newly diagnosed with endometriosis during this study.

One patient randomized to the control group accidently received no warming blanket, but warm humidified insufflation gas instead of the allocated intervention with forced air warming blanket alone. One patient randomized into group “HUMI” did not receive surgery and consequently the allocated intervention, because of a preoperative spontaneous rupture of the ovarian cyst that was the indication for laparoscopy. Three patients randomized into group “HUMI +” did not receive the allocated intervention: in two cases because the planned surgery was not performed due to different reasons and the other because the patient received a forced air warming blanket only instead of the allocated intervention.

In two cases the intervention was discontinued: one patient from group “AIR” because of the intraoperative indication to a conversion of the laparoscopic procedure into laparotomy and one patient from group “HUMI” because of the lowering of core body temperature below 35 °C during the intervention with the necessity to use in addition a forced air warming blanket.

Follow-up data were available for 146 of the 150 randomized patients. In one case from group “AIR” and one case from group “HUMI +”, follow-up data were missing, because the VAS questionnaires were not available. There were also no follow-up data from the two patients without surgery after randomization.

**Fig. 1:**
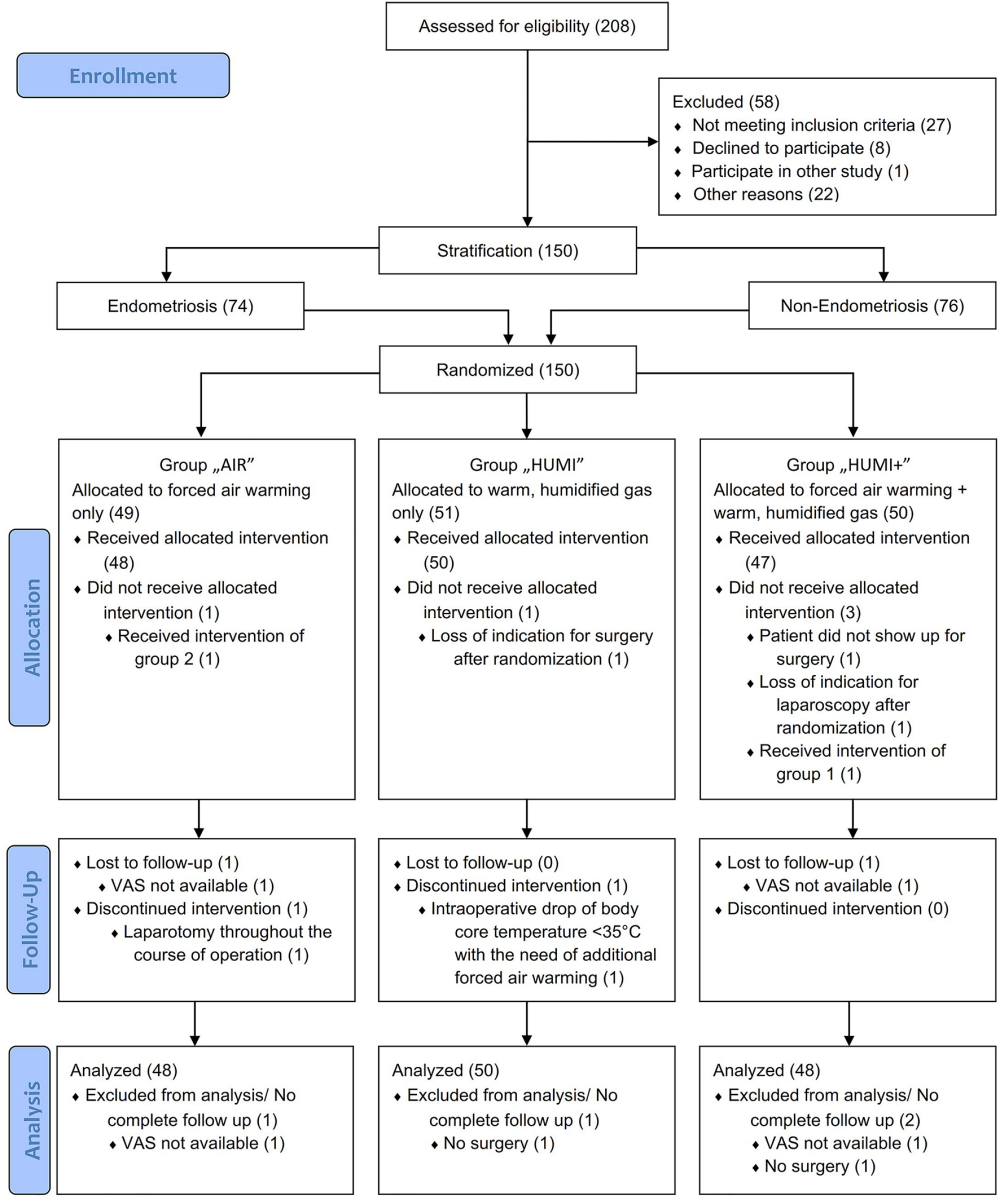
CONSORT flow diagram. Numbers are given in brackets

Primary intention-to-treat analysis was performed on the full set of follow-up data (Fig. 1).

### Baseline data

Table 1 shows the baseline characteristics of all patients who received surgery. Demographic data, risk factors for cardiovascular complications, patient’s medical history, intraoperative medication, IV fluids, and insufflated CO_2_, as well as the type and length of operative procedures were recorded. Significant differences between the groups were seen in ASA classification and intraoperative use of paracetamol. No other differences were recorded.

**Table 1:** Baseline characteristics of the investigated patient groups (all participants who underwent surgery)

	Group 1 “AIR” *n* = 49	Group 2 “HUMI” *n* = 50	Group 3 “HUMI +” *n* = 49	*p*-value
Age (years)	40.4 ± 14.0	36.1 ± 11.7	38.7 ± 11.6	0.51
BMI	24.7 ± 3.78	26.1 ± 4.71	23.0 ± 3.55	0.05
Smoker	16 (32.7)	18 (36.0)	7 (14.3)	
Cigarettes per day	13.5 ± 6.23	11.9 ± 6.82	11.1 ± 7.45	0.39
Smoking years	16.3 ± 14.5	16.2 ± 14.6	14.2 ± 7.6	0.79
Ex-smoker	8 (16.3)	11 (22.0)	14 (28.6)	
Cigarettes per day	12.5 ± 6.89	16.1 ± 6.97	11.5 ± 6.17	0.53
Smoking years	9.57 ± 7.91	11.30 ± 9.38	9.79 ± 6.89	0.96
Risk factors for CV complications				
Hypercholesterolaemia	3 (6.1)	2 (4.0)	3 (6.1)	0.82
Hypertension	9 (18.4)	5 (10.0)	4 (8.2)	0.26
Overweight	19 (38.8)	24 (48.0)	12 (24.5)	0.05
Co-morbidities				
Diabetes	3 (6.1)	1 (2.0)	3 (6.1)	0.57
Arteriosclerosis	1 (2.0)	0	0	0.66
Asthma	7 (14.3)	3 (6.0)	2 (4.1)	0.21
Thyroid dysfunction	7 (14.3)	14 (28.0)	9 (18.4)	0.22
ASA classification				0.04
ASA 1	19 (38.8)	22 (44.0)	33 (67.3)	
ASA 2	25 (51.0)	26 (52.0)	15 (30.6)	
ASA 3	4 (8.2)	2 (4.0)	1 (2.0)	
Not applicable	1 (2.0)			
Previous abdominal surgery				0.31
Laparoscopic	22 (44.9)	20 (40.0)	23 (46.9)	
Abdominal	12 (24.5)	12 (24.0)	6 (12.2)	
Epidural catheter	11 (28.2)	16 (41.0)	12 (30.8)	0.52
Anesthetics				
Propofol (mg/h)	419 ± 59.4 (n = 20)	437 ± 86.0 (n = 14)	387 ± 68.4 (n = 22)	0.10
Sevoflurane (%)	1.57 ± 0.290 (n = 28)	1.56 ± 0.285 (n = 37)	1.55 ± 0.213 (n = 22)	0.98
Desflurane (%)	5.00 (n = 1)	5.33 ± 0.808 (n = 3)	5.10 ± 0.316 (n = 5)	0.80
Intraoperative opioids				
Sufentanil (µg)	45.1 ± 15.1 (n = 47)	40.2 ± 13.0 (n = 50)	45.5 ± 18.3 (n = 48)	0.17
Remifentanil (µg/ h)	43 (n = 1)	1200 (n = 1)	(n = 0)	
Fentanil (mg)	0.5 ± 0.1 (n = 2)	(n = 0)	0.4 (n = 1)	
Piritramide (mg)	5.28 ± 2.28 (n = 29)	5.27 ± 2.33 (n = 32)	4.75 ± 2.15 (n = 23)	0.64
Intraoperative non-opioids				
Metamizole (g)	1.20 ± 0.391 (n = 23)	1.21 ± 0.379 (n = 26)	1.19 ± 0.385 (n = 24)	0.98
Paracetamol (g)	1 ± 0 (n = 8)	1 ± 0 (n = 1)	1 ± 0 (n = 4)	0.03
Ibuprofen (g)	(n = 0)	0.525 ± 0.125 (n = 2)	0.4 ± 0 (n = 2)	
Intraoperative relaxant				
Rocuronium (mg)	49.9 ± 21.1 (n = 49)	53.5 ± 23.7 (n = 50)	51.4 ± 19.7 (n = 49)	0.71
Length of anesthesia (min)	193 ± 104.0	187 ± 90.8	193 ± 94.2	0.94
Amount of infusions (ml)	1638 ± 796	1690 ± 748	1714 ± 661	0.87
Type of surgical procedure				
Endometriosis	19 (38.8)	24 (48.0)	20 (40.8)	0.62
Hysterectomy	12 (24.5)	14 (28.0)	18 (36.7)	0.39
Myoma enucleation	7 (14.3)	5 (10.0)	4 (8.2)	0.61
Cyst enucleation	8 (16.3)	8 (16.0)	4 (8.2)	0.41
Adnektomia	9 (18.4)	5 (10.0)	9 (18.4)	0.41
Other	13 (26.5)	19 (38.0)	18 (36.8)	0.42
Length of surgery (min)	169 ± 97.8	166 ± 84.6	171 ± 92.6	0.96
Amount of intraperitoneal irrigating fluids (ml)	849 ± 600	971 ± 597	976 ± 611	0.54
Length of capnoperitoneum (min)	109 ± 78.0	109 ± 77.1	116 ± 73.1	0.88
Amount of insufflated CO_2_ (l)	294 ± 338	261 ± 237	294 ± 229	0.79

Values are given as mean ± standard deviation or number (percent)

### Outcomes and estimation

#### Primary endpoint—Postoperative pain

**Fig. 2:**
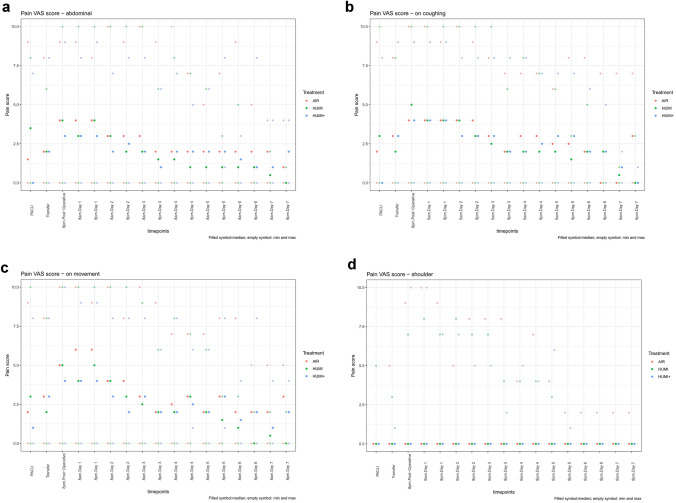
**A** VAS pain score of the three intervention groups—abdominal pain. “AIR” = red. “HUMI” = green. “HUMI +” = blue. (Filled symbol: median; empty symbol: min and max). **B** VAS pain score of the three intervention groups—pain on coughing. “AIR” = red. “HUMI” = green. “HUMI +” = blue. (Filled symbol: median; empty symbol: min and max). **C** VAS pain score of the three intervention groups—pain on movement. “AIR” = red. “HUMI” = green. “HUMI +” = blue. (Filled symbol: median; empty symbol: min and max). **D** VAS pain score of the three intervention groups—shoulder pain. “AIR” = red. “HUMI” = green. “HUMI +” = blue. (Filled symbol: median; empty symbol: min and max)

Descriptive statistics of pain perceived on each postoperative day specific for the abdominal area, shoulder pain, and pain caused by movement or coughing is shown in Table 2 and Fig. 2A–D. The maximum pain was evaluated to have a median of 6 in group “AIR” at day one during movement.

**Table 2:** VAS pain scores from admission to PACU until postoperative day seven

	Group 1 “AIR”	Group 2 “HUMI”	Group 3 “HUMI +”	*p*-value
Operation day				
Arrival in PACU				
VAS abdominal	1.5 (0 – 9)	3.5 (0 – 8)	0 (0 – 7)	0.13
VAS on coughing	2 (0 – 9)	3 (0 – 10)	0 (0 – 8)	0.31
VAS on movement	2 (0 – 9)	3 (0 – 10)	1 (0 – 8)	0.36
VAS shoulder pain	0 (0 – 0)	0 (0 – 5)	0 (0 – 0)	0.15
Transfer to ward				
VAS abdominal	2 (0 – 8)	2 (0 – 6)	2 (0 – 8)	0.86
VAS on coughing	3 (0 – 8)	2 (0 – 8)	3 (0 – 9)	0.68
VAS on movement	3 (0 – 8)	2 (0 – 8)	3 (0 – 8)	0.51
VAS shoulder pain	0 (0 – 5)	0 (0 – 3)	0 (0 – 1)	0.17
8 pm				
VAS abdominal	4 (0 – 9)	4 (0 – 10)	3 (0 – 9)	0.40
VAS on coughing	4 (0 – 10)	5 (0 – 10)	4 (0 – 9)	0.16
VAS on movement	5 (0 – 10)	5 (0 – 10)	4 (0 – 10)	0.21
VAS shoulder pain	0 (0 – 9)	0 (0 – 7)	0 (0 – 10)	0.69
Day 1				
8am				
VAS abdominal	4 (0 – 9)	3 (0 – 10)	3 (0 – 8)	0.74
VAS on coughing	4 (0 – 10)	4 (0 – 10)	4 (0 – 9)	0.46
VAS on movement	6 (0 – 10)	4 (0 – 10)	4 (0 – 9)	0.49
VAS shoulder pain	0 (0 – 10)	0 (0 – 8)	0 (0 – 10)	0.74
8 pm				
VAS abdominal	4 (0 – 8)	4 (0 – 10)	3 (0 – 9)	0.64
VAS on coughing	4 (0 – 9)	4 (0 – 10)	4 (0 – 9)	0.51
VAS on movement	6 (0 – 9)	5 (0 – 10)	4 (0 – 9)	0.42
VAS shoulder pain	0 (0 – 9)	0 (0 – 7)	0 (0 – 7)	0.15
Day 2				
8am				
VAS abdominal	3 (0 – 10)	3 (0 – 10)	2 (0 – 7)	0.17
VAS on coughing	4 (0 – 10)	4 (0 – 10)	3 (0 – 8)	0.35
VAS on movement	4 (0 – 10)	4 (0 – 10)	3 (0 – 8)	0.07
VAS shoulder pain	0 (0 – 5)	0 (0 – 8)	0 (0 – 7)	0.73
8 pm				
VAS abdominal	3 (0 – 9)	2 (0 – 10)	2.5 (0 – 8)	0.17
VAS on coughing	4 (0 – 9)	3 (0 – 10)	3 (0 – 8)	0.79
VAS on movement	4 (0 – 8)	3 (0 – 10)	2 (0 – 8)	0.04
VAS shoulder pain	0 (0 – 8)	0 (0 – 7)	0 (0 – 5)	0.89
Day 3				
8am				
VAS abdominal	3 (0 – 10)	2 (0 – 10)	2 (0 – 8)	0.11
VAS on coughing	3 (0 – 8)	2.5 (0 – 10)	3 (0 – 8)	0.63
VAS on movement	3 (0 – 10)	2.5 (0 – 9)	3 (0 – 8)	0.23
VAS shoulder pain	0 (0 – 8)	0 (0 – 7)	0 (0 – 5)	0.81
8 pm				
VAS abdominal	2 (0 – 9)	1.5 (0 – 6)	1 (0 – 6)	0.38
VAS on coughing	2 (0 – 7)	2 (0 – 6)	2 (0 – 8)	0.83
VAS on movement	2 (0 – 9)	2 (0 – 6)	2 (0 – 6)	0.37
VAS shoulder pain	0 (0 – 8)	0 (0 – 4)	0 (0 – 2)	0.80
Day 4				
8am				
VAS abdominal	2 (0 – 10)	1.5 (0 – 8)	2 (0 – 7)	0.45
VAS on coughing	3 (0 – 7)	2 (0 – 8)	2 (0 – 8)	0.29
VAS on movement	2.5 (0 – 7)	2 (0 – 8)	2 (0 – 8)	0.15
VAS shoulder pain	0 (0 – 4)	0 (0 – 4)	0 (0 – 5)	0.84
8 pm				
VAS abdominal	2 (0 – 7)	1 (0 – 7)	2 (0 – 5)	0.26
VAS on coughing	3 (0 – 7)	2 (0 – 7)	2.5 (0 – 7)	0.75
VAS on movement	3 (0 – 7)	3 (0 – 7)	2.5 (1 – 6)	0.55
VAS shoulder pain	0 (0 – 7)	0 (0 – 4)	0 (0 – 4)	0.32
Day 5				
8am				
VAS abdominal	2 (0 – 5)	1 (0 – 6)	2 (0 – 6)	0.15
VAS on coughing	2.5 (0 – 6)	2 (0 – 6)	3 (0 – 7)	0.87
VAS on movement	2 (0 – 7)	2 (0 – 6)	2 (0 – 6)	0.24
VAS shoulder pain	0 (0 – 4)	0 (0 – 3)	0 (0 – 6)	0.56
8 pm				
VAS abdominal	2 (0 – 7)	1 (0 – 3)	2 (1 – 8)	0.08
VAS on coughing	2.5 (0 – 8)	1.5 (0 – 3)	3 (0 – 7)	0.45
VAS on movement	3 (0 – 8)	1.5 (0 – 3)	3 (1 – 8)	0.20
VAS shoulder pain	0 (0 – 2)	0 (0 – 0)	0 (0 – 1)	0.10
Day 6				
8am				
VAS abdominal	2 (0 – 9)	1 (0 – 5)	1.5 (0 – 3)	0.26
VAS on coughing	2 (0 – 8)	2 (0 – 5)	2 (0 – 6)	0.96
VAS on movement	2 (0 – 8)	1 (0 – 3)	1.5 (0 – 4)	0.55
VAS shoulder pain	0 (0 – 2)	0 (0 – 0)	0 (0 – 0)	0.07
8 pm				
VAS abdominal	2 (0 – 5)	1 (0 – 1)	1 (0 – 8)	0.40
VAS on coughing	0 (0 – 7)	2 (0 – 2)	2 (0 – 5)	1
VAS on movement	2 (0 – 5)	0 (0 – 2)	2 (0 – 8)	0.45
VAS shoulder pain	0 (0 – 2)	0 (0 – 0)	0 (0 – 0)	0.37
Day 7				
8am				
VAS abdominal	2 (0 – 4)	0.5 (0 – 2)	1 (0 – 4)	0.47
VAS on coughing	0 (0 – 7)	0.5 (0 – 1)	1 (0 – 2)	0.87
VAS on movement	2 (0 – 5)	0.5 (0 – 2)	1 (0 – 4)	0.52
VAS shoulder pain	0 (0 – 2)	0 (0 – 0)	0 (0 – 0)	0.10
8 pm				
VAS abdominal	1 (0 – 4)	0 (0 – 1)	2 (0 – 4)	0.46
VAS on coughing	3 (0 – 7)	0 (0 – 3)	0 (0 – 1)	0.43
VAS on movement	3 (0 – 5)	0 (0 – 2)	2 (0 – 4)	0.44
VAS shoulder pain	0 (0 – 2)	0 (0 – 0)	0 (0 – 0)	0.37

Values are given as median (range)

*PACU* post-anesthesia care unit, *VAS* visual analogue scale

**Fig. 3:**
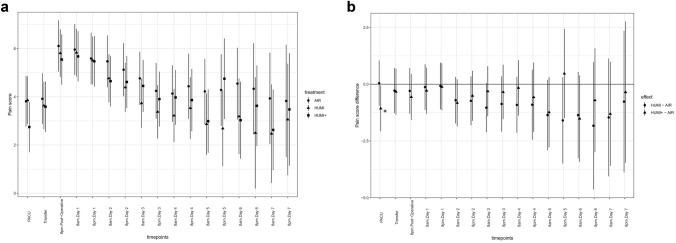
**A** Maximum pain scores of the three intervention groups. “AIR” = dot. “HUMI” = triangle. “HUMI +” = square. **B** Estimated treatment effect on pain score with significant less pain score in group “HUMI +” vs control group at arrival in PACU (Asterisk). “HUMI” vs “AIR” = dot. “HUMI +” vs “AIR” = triangle

Figure 3A shows the maximum pain score of the three treatment groups for each timepoint. Estimated treatment effects of the two interventional groups compared to the control group on postoperative pain scores are shown in Fig. 3B. The pain intensity upon arrival in PACU was significantly lower in group “HUMI +” as compared to group “AIR” (control group) (MD − 1.068; 95% CI − 2.08 to − 0.061), and there were no other differences between the groups. Also, after correcting for the effects of intraoperative analgesic use or the presence of epidural anesthesia in an additional explorative analysis, the difference in the pain score between group “HUMI +” and group “AIR” at arrival in PACU remained significant (MD − 1.068; 95% CI − 2.07 to − 0.069).

### Secondary endpoints

Table 3 shows a descriptive analysis of the secondary endpoints, like postoperative analgesic consumption, flow rate and duration of epidural anesthetics, length of stay in PACU, and length of the hospital stay. Compared to group “AIR”, group “HUMI +” showed significantly less consumption of Ibuprofen at day 2 (− 0.5871 g ± 0.258; *p*-value = 0.0471) (Table 4). No differences were found in the other secondary endpoints. Not shown are data of nausea and vomiting and ADL scores: there was no difference in occurrence of nausea and vomiting on day 1 (*p*-value 0.989) or on discharge day (*p*-value 0.6362). ADL scores were the same in each treatment group separately on day 1 (*p*-value 0.45) and on discharge day (*p*-value 0.2117).

**Table 3:** Secondary endpoints of the study

	Group 1 “AIR”	Group 2 “HUMI”	Group 3 “HUMI +”	*p*-value
Analgesic consumption				
Operation Day				
Piritramide (mg)	5.25 (3 – 24) [n = 24]	4.5 (3 – 22.5) [n = 31]	5.25 (1.5 – 22.5) [n = 26]	0.79
Metamizole (g)	1 (0.75 – 3) [n = 23]	1 (1 – 4) [n = 24]	1 (1 – 4) [n = 23]	0.48
Paracetamol (g)	1 (1 – 3) [n = 21]	1 (1 – 2) [n = 18]	1 (1 – 2) [n = 17]	0.43
Ibuprofen (g)	0.6 (0.4 – 1–8) [n = 8]	0.9 (0.4 – 1.2) [n = 4]	0.4 (0.4 – 1.2) [n = 8]	0.22
Analgesic consumption				
Day 1				
Piritramide (mg)	15 (3.75 – 22.5) [n = 6]	7.5 (3.75 – 18.8) [n = 12]	7.5 (3.75 – 15) [n = 9]	0.14
Metamizole (g)	2 (1 – 6) [n = 15]	2 (0.75 – 5) [n = 29]	2 (1 – 5) [n = 26]	0.95
Paracetamol (g)	1 (1 – 3) [n = 16]	1 (1 – 4) [n = 19]	2 (1 – 3) [n = 11]	0.45
Ibuprofen (g)	0.8 (0.4 – 2.0) [n = 11]	1.2 (0.6 – 2.4) [n = 9]	1.2 (0.8 – 3.0) [n = 11]	0.37
Analgesic consumption				
Day 2				
Piritramide (mg)	11.2 (3.75 – 18.8) [n = 2]	7.5 (7.5 – 7.5) [n = 2]	7.5 (7.5 – 15) [n = 3]	0.90
Metamizole (g)	1 (0.5 – 4) [n = 17]	2 (1 – 4) [n = 18]	2.25 (0.5 – 4) [n = 16]	0.26
Paracetamol (g)	1 (1 – 3) [n = 5]	1 (1 – 3) [n = 14]	2 (1 – 3) [n = 7]	0.57
Ibuprofen (g)	1.7 (0.4 – 4.0) [n = 10]	0.8 (0.4 – 2.4) [n = 10]	1.2 (0.4 – 1.8) [n = 14]	0.20
Analgesic consumption				
Day 3				
Piritramide (mg)	15 (3.75 – 26.2) [n = 2]	[n = 0]	22.5 (22.5 – 22.5) [n = 1]	
Metamizole (g)	2 (0.5 – 4) [n = 11]	2 (1 – 4) [n = 10]	2 (1 – 4) [n = 13]	0.80
Paracetamol (g)	[n = 0]	2 (1 – 3) [n = 5]	1.5 (1 – 2) [n = 2]	
Ibuprofen (g)	1.4 (0.4 – 2.8) [n = 12]	0.6 (0.4 – 2.4) [n = 7]	1.2 (0.4 – 1.8) [n = 7]	0.23
Analgesic consumption				
Day 4				
Piritramide (mg)	7.5 (7.5 – 7.5) [n = 1]	3.75 (3.75 – 3.75) [n = 1]	7.5 (7.5 – 7.5) [n = 1]	
Metamizole (g)	1.5 (1 – 4) [n = 5]	2 (1 – 4) [n = 7]	2 (0.5 – 5) [n = 12]	0.75
Paracetamol (g)	[n = 0]	2 (2 – 2) [n = 3]	1 (1 – 1) [n = 1]	
Ibuprofen (g)	1.4 (0.8 – 1.8) [n = 10]	1.2 (1.2 – 1.8) [n = 3]	1.2 (0.4 – 2.4) [n = 6]	0.10
Analgesic consumption				
Day 5				
Piritramide (mg)	[n = 0]	3.75 (3.75 – 3.75) [n = 1]	3.75 (3.75 – 3.75) [n = 1]	
Metamizole (g)	1 (0.5 – 5) [n = 3]	3 (1 – 4) [n = 6]	2 (1 – 4) [n = 7]	0.74
Paracetamol (g)	1 (1 – 1) [n = 2]	2 (2 – 2) [n = 1]	[n = 0]	
Ibuprofen (g)	1.2 (0.4 – 1.8) [n = 9]	1.5 (1.2 – 1.8) [n = 2]	1.5 (0.8 – 2.4) [n = 4]	0.83
Analgesic consumption				
Day 6				
Piritramide (mg)	[n = 0]	[n = 0]	[n = 0]	
Metamizole (g)	2 (1 – 3) [n = 2]	4 (2 – 4) [n = 3]	2 (2 – 4) [n = 3]	0.45
Paracetamol (g)	1 (1 – 1) [n = 1]	[n = 0]	1 (1 – 1) [n = 1]	
Ibuprofen (g)	1.2 (0.8 – 4.0) [n = 7]	1.5 (1.2 – 1.8) [n = 2]	1.2 (1.2 – 1.2) [n = 1]	0.63
Analgesic consumption				
Day 7				
Piritramide (mg)	[n = 0]	[n = 0]	[n = 0]	
Metamizole (g)	3 (3 – 3) [n = 1]	4 (4 – 4) [n = 1]	2 (1 – 4) [n = 3]	
Paracetamol (g)	[n = 0]	[n = 0]	1 (1 – 1) [n = 1]	
Ibuprofen (g)	1.2 (0.8 – 1.6) [n = 3]	1.5 (1.2 – 1.8) [n = 2]	0.8 (0.8 – 0.8) [n = 1]	
Epidural anesthesia				
Duration (hours)	70 (31.5 – 145)	53.2 (5 – 118)	58.5 (4.5 – 123)	0.39
Flow rate (ml/h)	4 (3 – 6)	4.73 (3.43 – 6)	5.17 (3.2 – 6)	0.22
Length of stay				
In PACU (mins)	88 (27 – 318)	95 (10 – 270)	90 (30 – 235)	0.96
In hospital (days)	4.5 (1 – 10)	4.5 (0.5 – 14)	4.75 (1 – 13.5)	0.94

Values are given as median (range)

**Table 4:** Estimated treatment effects on secondary endpoints with significant less consumption of Ibuprofen in group “HUMI +” vs control group at day 2 (Asterisk)

	“HUMI” – “AIR”		“HUMI +” – “AIR”	
	Estimated effect	*p*-value	Estimated effect	*p*-value
Operation day				
Piritramide (mg)	− 0.997 ± 1.60	0.7479	− 1.525 ± 1.66	0.5589
Metamizole (g)	0.3507 ± 0.340	0.4859	0.1735 ± 0.343	0.8192
Paracetamol (g)	− 0.174 ± 0.214	0.6246	− 0.117 ± 0.217	0.7984
Ibuprofen (g)	0.1479 ± 0.372	0.8780	− 0.2145 ± 0.302	0.6928
Day 1				
Piritramide (mg)	−6.221 ± 3.01	0.0767	−5.485 ± 3.16	0.1549
Metamizole (g)	−0.0527 ± 0.367	0.9778	0.0784 ± 0.374	0.9580
Paracetamol (g)	0.340 ± 0.226	0.2370	0.359 ± 0.261	0.2939
Ibuprofen (g)	0.0478 ± 0.276	0.9695	0.2236 ± 0.265	0.6060
Day 2				
Piritramide (mg)	−4.037 ± 6.09	0.7243	−2.403 ± 5.57	0.8607
Metamizole (g)	0.5944 ± 0.387	0.2229	0.4153 ± 0.397	0.4763
Paracetamol (g)	0.106 ± 0.348	0.9219	0.344 ± 0.391	0.5829
Ibuprofen (g)	−0.6162 ± 0.275	0.0508	−0.5871 ± 0.258	0.0471*
Day 3				
Piritramide (mg)	NA	NA	4.537 ± 7.52	0.7616
Metamizole (g)	−0.0536 ± 0.481	0.9855	0.2940 ± 0.455	0.7341
Paracetamol (g)	NA	NA	NA	NA
Ibuprofen (g)	-0.5914 ± 0.291	0.0828	-0.3248 ± 0.291	0.4349
Day 4				
Piritramide (mg)	−3.037 ± 8.49	0.8974	−1.764 ± 8.69	0.9603
Metamizole (g)	0.7638 ± 0.631	0.3800	0.1301 ± 0.575	0.9522
Paracetamol (g)	NA	NA	NA	NA
Ibuprofen (g)	−0.3667 ± 0.391	0.5458	−0.2056 ± 0.311	0.7245
Day 5				
Piritramide (mg)	NA	NA	NA	NA
Metamizole (g)	0.7954 ± 0.761	0.4774	0.0297 ± 0.738	0.9973
Paracetamol (g)	0.843 ± 0.816	0.4856	NA	NA
Ibuprofen (g)	0.0509 ± 0.465	0.9859	0.1813 ± 0.355	0.8161
Day 6				
Piritramide (mg)	NA	NA	NA	NA
Metamizole (g)	1.4072 ± 0.952	0.2473	1.1877 ± 0.948	0.3569
Paracetamol (g)	NA	NA	0.000 ± 0.940	1.000
Ibuprofen (g)	−0.0916 ± 0.475	0.9636	−0.1686 ± 0.588	0.9290
Day 7				
Piritramide (mg)	NA	NA	NA	NA
Metamizole (g)	0.2778 ± 1.432	0.9632	−0.8085 ± 1.184	0.7106
Paracetamol (g)	NA	NA	NA	NA
Ibuprofen (g)	0.2369 ± 0.534	0.8536	−0.2402 ± 0.636	0.8879
Epidural anesthesia duration (hours)	−23.4 ± 15.7	0.2534	−14.2 ± 16.7	0.6058
Length of stay in PACU (mins)	−1.810 ± 10.316	0.861	−2.930 ± 10.245	0.775

### Ancillary analyses

Additionally, exploratory post hoc analysis of postoperative pain scores was performed. We analyzed the treatment effect on postoperative pain scores of the two intervention groups combined, both received warm and humidified gas, and in comparison to the control group, which received cold and dry gas: no significant differences in this model were detected.

**Fig. 4:**
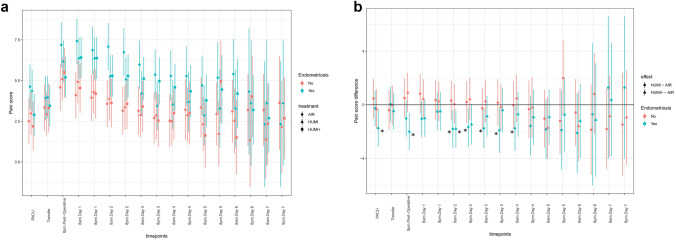
**A** Maximum pain scores of the three intervention groups with stratification by endometriosis. “AIR” = dot. “HUMI” = triangle. “HUMI +” = square. **B** Estimated treatment effect on pain score with stratification by endometriosis. Significant less pain score in subjects suffering from endometriosis (Asterisk). “HUMI” vs “AIR” = dot. “HUMI +” vs “AIR” = triangle

Results stratified by endometriosis are shown in Fig. 4A and B. Figure 4A shows pain scores stratified by endometriosis with higher pain scores in patients suffering from endometriosis nearly in all groups over the whole period. The differences between groups “HUMI” vs “AIR” and “HUMI +” vs “AIR” are shown in Fig. 4B. There were significantly lower pain score levels of subjects suffering from endometriosis in group “HUMI” and group “HUMI +” compared to group “AIR” at different timepoints.

**Fig. 5:**
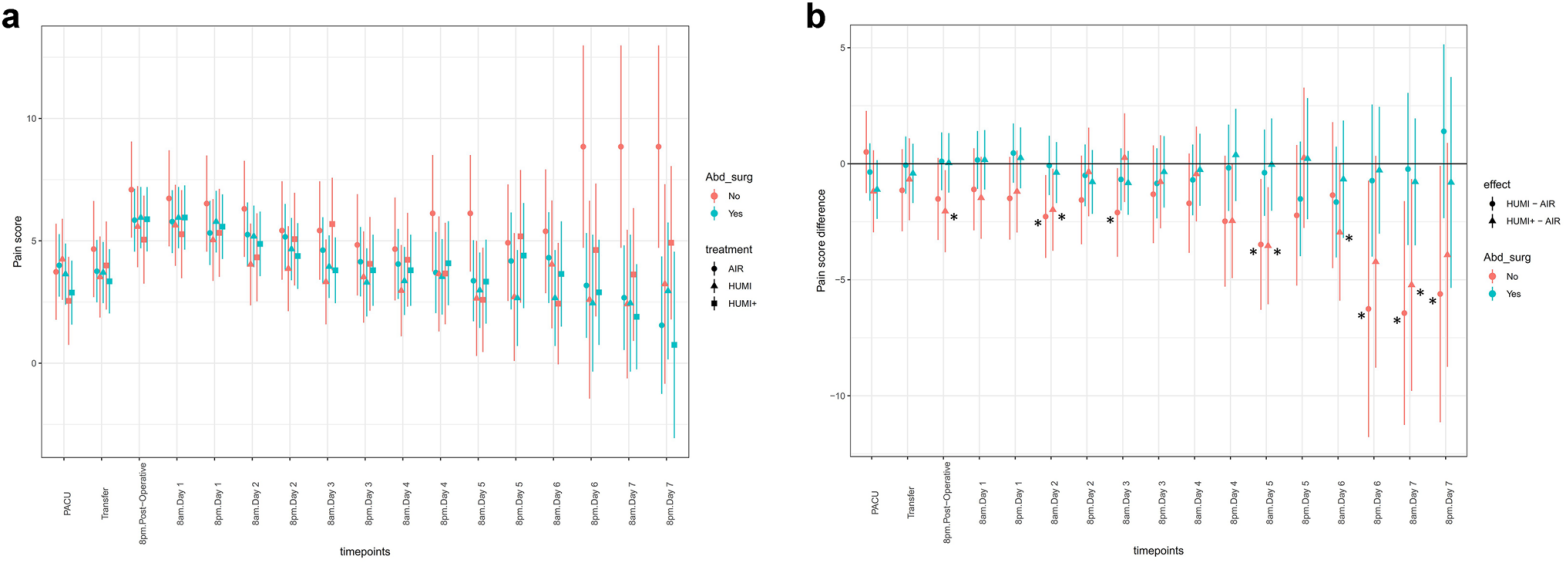
**A** Maximum pain scores of the three intervention groups with stratification by previous abdominal surgery. “AIR” = dot. “HUMI” = triangle. “HUMI +” = square. **B** Estimated treatment effect on pain score with stratification by previous abdominal surgery. Significant less pain score in subjects, without previous abdominal surgery (Asterisk). “HUMI” vs “AIR” = dot. “HUMI +” vs “AIR” = triangle

Analysis of data stratified by abdominal surgery showed significantly less pain scores in patients who did not undergo previous abdominal surgery in both groups “HUMI” and “HUMI +” compared to group “AIR” at different timepoints (Fig. 5A and B).

### Adverse events

A total of 109 (74%) study objects experienced adverse events during study intervention. All adverse events were classified as mild and they were not, or highly unlikely to be, related to the study (see Supplement, Table S1).

## Discussion

In the present study, we investigated the effect of three different intraoperative management regimens using either standard conditions with a heating blanket with forced air warming and dry insufflation gas at room temperature (group “AIR”), no heating blanket, but warm and humidified insufflation gas (group “HUMI”) or both a heating blanket with forced air warming and warm and humidified insufflation gas (group “HUMI +”). Importantly, we could not detect clinically relevant differences over the whole patient population. We could work out that patients suffering from endometriosis and patients without previous abdominal surgery profited from the use of humidified insufflation gas.

Although reduced in comparison to open surgery, pain after laparoscopic surgery is a common phenomenon exceeding the pure nociception by wounds, drains, or sore intra-abdominal tissue. Knowledge about “head zones” is well described in several pathologies, and the occurrence of shoulder pain after laparoscopic surgery is common. Some studies have indicated that the use of dry insufflation gas to establish the capnoperitoneum might increase the nociception or, in fact, induce an inflammatory reaction of the peritoneal tissue [11–15].

In our study, we could not detect differences in postoperative pain in groups “HUMI” and “HUMI +” versus group “AIR” besides the timepoint “arrival at PACU” between group “HUMI +” and group “AIR.” The actual VAS score for abdominal pain in group “HUMI +” was 0 (0;7) and 1.5 (0;9) in group “AIR” at this timepoint; therefore, we interpret this significant difference as an effect of direct individual anesthesia but not of the intervention. Interestingly, there is no difference in the amount of postoperative analgesic medication (in our hospital, mainly piritramide combined with a non-opioid analgesic) which could explain this difference. Moreover, the use of analgesics on the day of surgery did not differ significantly, which could have been expected, if pain was higher in groups “AIR” and “HUMI.” Additionally, the effects were not altered when combining the groups receiving warm and humidified gas with and without the intraoperative use of a heating blanket (group “HUMI” and “HUMI +”) versus the control group, where cold and dry insufflation gas was applied (group “AIR”).

At day 2, we measured less consumption of Ibuprofen in group “HUMI +” compared to group “AIR,” while the effect did not occur in group “HUMI” versus group “AIR”; no other non-opioid was used differently that could have replaced ibuprofen in the other group. This difference may simply be due to the preferences of the treating physician or nurse on this day and, in our opinion, should not lead to the conclusion that group “HUMI +” really experienced less pain: in fact, in all patient groups, the VAS at day 2 was moderate with a VAS of 3 at rest and not significantly different.

These findings are in line with Matsuzaki and her colleagues’ work that could not find differences between the likelihood of higher pain intensity (VAS > 3) in the PACU and a significantly higher use of opioid analgesics [29]. This study, however, detected a higher likelihood of pain in the first 12 h after surgery in their 2 × 2 mixed model of high intra-abdominal pressure with or without WHCD and low intra-abdominal pressure with or without WHCD, when dry gas was used. In a comparable setting, Herrmann et al. reported in their study significantly less shoulder tip pain at 6 h, when all VAS points were cumulated over 48 h: the actual VAS scores were very low in both groups (in the mean at 6 h 0.09 vs 0.45 in the control group), so that the clinical relevance might be reduced [30]. Other clinical studies demonstrate conflicting results [31, 32] or describe a reduction in shoulder tip pain only [33]. In a recent meta-analysis with a mixed patient population (surgical, gynecological), there could be evidence for reduced pain in the first 8 h after surgery and less morphine use; interestingly, the underlying studies with clear benefits for humidified gas in this meta-analysis were surgical interventions (bariatric and cholecystectomy), while the gynecological studies included did not show benefits for humidified gas [17]. Otherwise, the lack of a difference between group “HUMI” and “AIR” may justify using warmed, humidified gas as standard instead of forced air warming, due to a physician’s choice or for possible economic reasons.

Considering that epidural anesthesia in some patients may have influenced our results, we performed the exploratory analysis to correct for this effect without major influence on the results: in fact, group “HUMI +” had less pain in PACU, but this effect did not extend through the next hours or days. Epidural anesthesia is a standard in a wide field of indications, even in laparoscopic surgery, and yields impressive results [34]. However, for simple hysterectomy, the technique may be too invasive, whereas in patients with endometriosis, it is a helpful tool to reduce pain in a predisposed patient population suffering from pain, often for years. In fact, it did not influence the results of our intervention.

### Insufflation of humidified gas in predisposed patient groups

Our post hoc analysis revealed two important results. First, patients suffering from endometriosis showed higher pain score levels than non-endometriosis patients nearly over the whole period during our observation (Fig. 4A). However, we detected significantly less pain in both intervention groups compared to the control group if endometriosis was present, which lasted for several days (Fig. 4B). This is a novel finding, indicating that female patients suffering from endometriosis may especially profit from the use of humidified and warm insufflation gas, while generally in higher pain. This can have several possible explanations. First, patients with endometriosis may be suffering from chronic pain, which often causes structural and functional changes in the nociceptive system. Endometriosis induces inflammation in the tissue surrounding it [35] and can be found in 70% of patients suffering from chronic pelvic pain [36]. Additionally, the surgery characteristics may also influence the results of the study. For instance, surgical treatment of endometriosis, which is, according to our clinic standards, performed via excision of the affected areas, results in the stripping of peritoneum and exposure of relatively large surfaces of underlying tissue to the insufflation gas, possibly facilitating tissue drying and other local reactions. This differs from other gynecologic surgeries, like hysterectomy, myomectomy, or ovarian cystectomy, where the exposed surface can be smaller or covered by coagulation area or surgical sutures.

When grouping our patients according to prior or non-prior abdominal surgery, we measured a higher pain level in patients without pre-existent surgery (Fig. 5A), which may be due to the fact, that in pre-operated patients with a likelihood for adhesions in 40–63% of gynecological or obstetric patients, [37] pain relief by adhesiolysis may have been beneficial per se [38, 39]. Nonetheless, the use of WHCD leads to a significant reduction of pain in the patient group facing higher pain levels. Therefore, patient groups with the risk of higher postoperative pain (in our study endometriosis patients and those without prior surgery) may potentially benefit from using warm humidified insufflation gas, which prevents the drying of the wound surfaces during surgery that may contribute to intraoperative pain [7]. In rodent experiments, the use of humidified gas protected against both adhesions and the surface reaction of mesothelium and peritoneum was ameliorated compared to dry gas [11, 12]. In porcine models, the authors describe an increase in peritoneal damage simply using dry gas; these studies demonstrated further that HIF-1α, a string indicator for tissue hypoxia but also a modulator in pain regulation, was enhanced [15, 40]. Additionally, HIF-1α disturbances have been revealed to be present in endometriosis formation and reaction [41]. We did not measure HIF-1α in tissue or abdominal fluid, etc. to proof this theory, but it may be of interest for further studies investigating effects on abdominal pain by insufflation gas.

### Limitations and strength

Our study has several strengths and limitations that need to be addressed. First, the study design had a clear randomization, single blinding, a pre-defined surgical team, and a variety of measures to assess “pain” in a well-defined homogeneous population.

Since the study was designed to address heating capabilities and pain reduction, three groups were constructed, although for the research question, if humidified and warm insufflation gas reduces postoperative pain, a two-sided model would have been sufficient. In our analysis, we remained in the group “HUMI”/ “AIR” and “HUMI +”/ “AIR” design; one model was calculated with both groups pooled against group “AIR” without changing significance levels and under consideration of statistical balancing. Because we did not see any differences, we remained in the three-group model to follow the investigation plan properly.

This study was powered to detect a difference in intraoperative core body temperature. As a limitation, we must mention that no power was calculated for questioning pain score differences.

The unequal distribution of patients stratified by previous abdominal surgery is worth mentioning. While the endometriosis/ non-endometriosis patients are nearly equally distributed, there are 66% with previous abdominal surgery and only 1/3 without. Therefore, we must assume that the results stratified by previous abdominal surgery are perhaps less meaningful.

It is of interest whether the results of this monocentric study can be extrapolated to other patient populations (e.g., urologic) or reproduced in other hospital settings.

Further studies on the pathogenetic mechanisms of the observed differences, involving inflammatory pathways and HIF-1α, especially in the subgroup of patients with endometriosis, are needed.

The adverse events we recorded were overall mild and not related to the study and unlikely explainable simply by the use of differently warm and humid insufflation gas.

## Conclusion

Application of prewarmed and humidified insufflation gas during laparoscopic surgery was not clinically relevant in reducing post-surgical pain in a mixed gynecological patient population. However, patients suffering from endometriosis or patients with expected high pain levels, in our study patients without a history of abdominal surgery, showed less pain up to several days. Therefore, in predisposed patients the use of preheated and humidified insufflation gas may be beneficial.

## Supplementary Information

Below is the link to the electronic supplementary material.

Supplementary file1 (DOCX 103 kb)
