# Supplementary material for: Warm and humidified insufflation gas during gynecologic laparoscopic surgery reduces postoperative pain in predisposed patients—a randomized, controlled multi-arm trial
Source: Surg Endosc. 2021 Oct 1;36(6):4154–70. doi: 10.1007/s00464-021-08742-1 (PMC9085687; doi:10.1007/s00464-021-08742-1)
Supplement: Supplementary file 1 — Supplementary file1 (DOCX 103 kb) [file 464_2021_8742_MOESM1_ESM.docx]

Supplement 1

Table S1: list of adverse events in interventional and control groups.

## Supplement 2 detailed methods description

#### Trial design

The study was designed as a monocentric, prospective, randomized, double-blinded controlled trial with three parallel intervention arms. Before trial commencement, the study design was changed into a single-blinded trial because the surgeons and study staff could not be effectively blinded with respect to the devices used during the laparoscopic procedure. All patients and ward staff were not aware of the method used during laparoscopy.

#### Ethic committee and trial registration

The study was approved by the Ethics Committee at the RWTH Aachen University Faculty of Medicine, Germany, in August 2015. The trial is registered under the name “Temperature and Pain in Laparoscopy” (TePaLa) with ClinicalTrials.gov on May 17, 2016, trial number NCT02781194.

#### Participants

The study included 150 participants with an indication to a laparoscopic gynecological surgery. It was conducted at the Department of Anesthesiology and the Department of Gynecology and Obstetrics, University Hospital Aachen, Germany between July 2016 and September 2018.

The participants were randomized in 3 groups of 50 subjects each. In group „AIR“ (control group „AIR”), a forced air warming blanket and cold and dry insufflation gas was used during surgery. In group „HUMI“, insufflation was performed with warm and humidified insufflation gas, and no warming blanket was used („HUMI“). Group „HUMI+“ was treated with a combination of a forced air warming blanket and warm, humidified gas („HUMI+“).

#### Inclusion criteria

Eligible patients were female, aged between 18 to 69 years with a body mass index under 35, admitted to the hospital for laparoscopic surgery with a planned duration of more than 60 minutes.

#### Exclusion criteria

Exclusion criteria were patients who were pregnant or not using sufficient contraception, who were breastfeeding, who were engaged in alcohol or drug abuse, who were either expected not to comply with instructions or with limited ability to comply with instructions for this study, who were unwilling or unable to give informed consent, who participated in another interventional study within the last 3 months, who are committed to an institution and/ or penitentiary by judicial or official order. and who are employees of the investigator cooperation companies.

The study could have been terminated due to a violation of the study protocol, the declaration of Helsinki, ICH-GCP, and/ or applicable regulatory requirements. Participants of the study were excluded if the patient withdrew informed consent or did not follow instructions by the study team, if their safety and well-being could not be ensured any longer, if the risks and benefits of continuing the study were reassessed and the risk outweighed any potential benefit, if the incidence of adverse events (AE) constituted a potential health hazard to the subjects, if perioperative temperature dropped below 35.0°C (core body temperature), or if intraoperative loss of blood was more than 500ml.

After a physical examination and elucidation about the study course by a senior physician, eligible patients were requested to participate in the study.

#### Interventions

#### Pre-surgical treatment

Patients were warmed with a duvet for one hour before the procedure in the holding area of operation theatres. An underbody blanket for delivery of forced air warming was placed on every procedure table before patients were placed on it but was used only in groups „AIR“ and „HUMI+“. This ensured allocation concealment for patients and provided a possibility of external warming up in case the core body temperature dropped below 35°C in group „HUMI“ (exclusion criterion). During transfer from the holding area to the operation room, all patients were covered with prewarmed cotton sheets. The ambient temperature of anesthetic preparation room and operation theatre was 21°C, measured and set by the central air conditioning system.

#### Intra-operative procedures

If epidural anesthesia was indicated and desired by the patient, an epidural catheter was placed according to standard operating procedures. On the day of surgery and on the first postoperative day, epidural anesthesia was performed using a mixture of ropivacaine 0.3% and sufentanil 0.75 µg/ml. Afterwards, ropivacaine 0.2% was admitted without sufentanil. All patients received general anesthesia as total intravenous anesthesia or low flow (<1l/ min) balanced anesthesia. After the induction of anesthesia, patients of group „AIR“ and group „HUMI+“ received forced air warming, administered by the 3M Bair Hugger Warming Unit, Model 775 in combination with 3M Bair Hugger Lithotomy Underbody Blanket, Model 585 (both 3M company, St Paul, USA). Patients of group „HUMI“ were only covered with cotton sheets. All participants underwent laparoscopic surgery in a lithotomy position, which was performed by one of four surgeons appointed by the principal investigator because of their similar operating techniques to avoid side effects on postoperative pain. According to randomization, capnoperitoneum was established and maintained either with cold and dry CO_2_ (21.0°C room temperature/ 0% humidity) in group „AIR“ or with warm and humidified CO_2_ (depending on flow rate > 38.6°C/ > 98%)[22] in group „HUMI“ and group „HUMI+“. Insufflation gas was warmed and humidified by the F&P HumiGard Surgical Humidifier MR860 (Fisher & Paykel Healthcare Limited, Auckland, New Zealand). Heated and humidified gas was supplied by the ST310 Laparoscopic Humidification Kit (Fisher & Paykel Healthcare Limited, Auckland, New Zealand). The actively heated tube maintained the temperature and humidity of the gas until it was delivered to the patient interface (37.0°C ± 0.8/ 100.0% ± 0.05)[23]. For CO_2_ insufflation, 26432020-1 THERMOFLATOR from KARL STORZ was used (KARL STORZ SE & Co. KG, Tuttlingen, Germany). Maximum gas pressure was set to 15 mmHg, so that except for the laparoscopic port entry procedure, the maximum intraperitoneal pressure did not exceed this limit. Fluid management contained an intravenous fluid input of minimum 4ml/ kg/ h with an average aim of 500ml/ h. Intravenous fluids were administered via HL-90-DE-230 HOTLINE Blood and Fluid Warmer (Smiths Medical, Inc., Minneapolis, USA) in all groups. Prior to the end of surgery, all patients received Metamizole IV (10 – 20 mg/ kg KG) or Paracetamol IV (1g), in case of hypersensitivity to Metamizole, and Piritramide IV (0.05 – 0.1 mg/ kgKG) 20 – 30 minutes before skin closure.

#### Post surgical data acquisition

After the patient’s arrival in PACU, the pain score was determined with the visual analogue scale (VAS) for pain from the abdominal area, pain in the shoulder, pain upon movement and pain upon coughing. Pain scores were also recorded before transfer to the ward, on the day of surgery at 8 p.m. and on postoperative days 1 to 7 at 8 a.m. and 8 p.m. until the day of the patient’s dismissal from the hospital. All patients were instructed to use VAS on the day before surgery, and the pain questionnaire was filled by the patient alone to avoid observer bias. Postoperative pain management was standardized and followed a three-step analgesic ladder, based on the WHO guidelines for the pharmacological and radiotherapeutic management of cancer pain in adults and adolescents [24]. Metamizole, paracetamol, or ibuprofen were used as non-opioid, and piritramide as opioid analgesics. Demand of non-opioid and opioid painkillers was recorded by ward staff until discharge day. All patients underwent a survey by the study staff one day before surgery, on the first postoperative day, and on the day of discharge to collect the data on nausea and/ or vomiting as well as ADL scores.

#### Outcomes

The primary endpoint was postoperative pain recorded by the visual analogue scale upon arrival in the recovery room, before transfer to the ward, at 8 p.m. on the day of surgery, at 8 a.m. and 8 p.m. on postoperative days 1 to 7 specifically for abdominal pain, pain in the shoulders, pain upon movement and pain upon coughing. Secondary endpoints were analgesic consumption, duration of epidural anesthesia, postoperative nausea and vomiting, differences in activities of daily living (ADL), the length of stay in post anesthesia care unit (PACU), and the total length of the hospital stay.

#### Sample size

This study was designed to address heating capabilities and pain reduction. Three groups were constructed, and sample size and statistical power were calculated to detect a difference in core body temperature. In a Cohen´s delta effect size power analysis, a sample size of 50 in each of the treatment groups would give a power of 0.8 to detect a difference of at least 0.2°C between groups in a balanced design. The effects on body temperature have not been published yet.

#### Randomization

After patients were enrolled by the study team and written informed consent was obtained, study participants were randomized with equal allocation ratios to the three interventions using permuted block randomization (block size 6) stratified by endometriosis (Yes/No). Computer generated sequences were used. To maintain allocation concealment, the randomization sequence and the block size were concealed from the investigators and the study team until database lock, and the assignment to study participants was carried out with a web-based application maintained by the Institute of Medical Informatics, RWTH Aachen University.

#### Statistical methods

Outcome variables were described within each treatment group using standard descriptive statistics (frequency, minimum, maximum, quartiles, mean, and standard deviation). Descriptive statistics for pain scores were also calculated separately for each measurement time point. Analyses of pain scores were performed on the maximum pain score calculated as the maximum of the abdominal-, movement-, on coughing- and shoulder pain scores for each subject at each measurement occasion. A linear mixed effects model was used to model pain score [25,26]. The model included fixed effects for the treatments, measurement occasions, stratification, and randomization blocks, as well as measurement-treatment interactions. The random part included intercepts grouped by subjects. We based the inference on the treatment effect on a likelihood-ratio test comparing the full model with a restricted model excluding all treatment effects. Estimated treatment effects at each measurement occasion were calculated from the model along with nominal 95% confidence intervals. Additional explorative analyses were conducted with adjustment for analgesic use (yes/no) and the presence of an epidural catheter (yes/no). Analgesic use was defined as Piritramide > 0.05 mg/kg, or Metamizole > 2g, or Paracetamol > 2g, or Ibuprofen > 800mg. Explorative tests for the treatment effect on activities of daily living (ADL) scores and the frequency of nausea severity levels on day 1 and at discharge were conducted with Kruskal-Wallis rank sum test and Pearson’s Chi-squared tests, respectively. The length of stay in PACU was analyzed using a general linear model with gamma distributed errors. Analyses were conducted using R [27]. Mixed models were fitted with lme4 [28].
